# Cu-doped calcium phosphate supraparticles for bone tissue regeneration†

**DOI:** 10.1039/d4ra04769a

**Published:** 2024-10-17

**Authors:** Anika Höppel, Olivia Bahr, Regina Ebert, Annette Wittmer, Michael Seidenstuecker, M. Carolina Lanzino, Uwe Gbureck, Sofia Dembski

**Affiliations:** a Department Tissue Engineering and Regenerative Medicine (TERM), University Hospital Würzburg 97070 Würzburg Germany anika.hoeppel@isc-extern.fraunhofer.de; b Department of Musculoskeletal Tissue Regeneration, University of Würzburg 97074 Würzburg Germany; c Medical Center University of Freiburg, Faculty of Medicine, Institute for Microbiology and Hygiene 79104 Freiburg Germany; d G.E.R.N. Center of Tissue Replacement, Regeneration & Neogenesis, Department of Orthopedics and Trauma Surgery, Faculty of Medicine, Albert-Ludwigs-University of Freiburg 79106 Freiburg Germany; e Institute for Manufacturing Technologies of Ceramic Components and Composites (IFKB), University of Stuttgart 70569 Stuttgart Germany; f Department for Functional Materials in Medicine and Dentistry, University of Würzburg 97070 Würzburg Germany; g Fraunhofer Institute for Silicate Research ISC 97082 Würzburg Germany sofia.dembski@isc.fraunhofer.de

## Abstract

Calcium phosphate (CaP) minerals have shown great promise as bone replacement materials due to their similarity to the mineral phase of natural bone. In addition to biocompatibility and osseointegration, the prevention of infection is crucial, especially due to the high concern of antibiotic resistance. In this context, a controlled drug release as well as biodegradation are important features which depend on the porosity of CaP. An increase in porosity can be achieved by using nanoparticles (NPs), which can be processed to supraparticles, combining the properties of nano- and micromaterials. In this study, Cu-doped CaP supraparticles were prepared to improve the bone substitute properties while providing antibacterial effects. In this context, a modified sol–gel process was used for the synthesis of CaP NPs, where a Ca/P molar ratio of 1.10 resulted in the formation of crystalline β-tricalcium phosphate (β-TCP) after calcination at 1000 °C. In the next step, CaP NPs with Cu^2+^ (0.5–15.0 wt%) were processed into supraparticles by a spray drying method. Cu release experiments of the different Cu-doped CaP supraparticles demonstrated a long-term sustained release over 14 days. The antibacterial properties of the supraparticles were determined against Gram-positive (*Bacillus subtilis* and *Staphylococcus aureus*) and Gram-negative (*Escherichia coli*) bacteria, where complete antibacterial inhibition was achieved using a Cu concentration of 5.0 wt%. In addition, cell viability assays of the different CaP supraparticles with human telomerase-immortalized mesenchymal stromal cells (hMSC-TERT) exhibited high biocompatibility with particle concentrations of 0.01 mg mL^−1^ over 72 hours.

## Abstract 2

Antibacterial, biodegradable spray dried Cu-doped CaP supraparticles with high cell viability show great potential as bone substitute materials.

## Graphical abstract

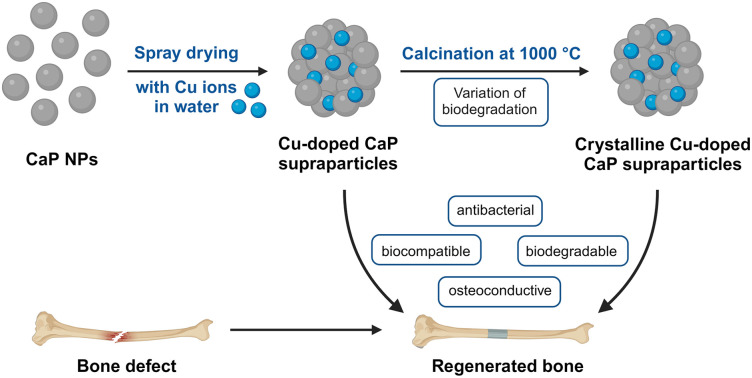

## 1 Introduction

Calcium phosphates (CaPs) are widely applied in the field of bone tissue regeneration due to their high biocompatibility, osteoconductivity and osseointegration.^1,2^ There are a variety of CaP compounds, distinguished by their stoichiometry, of which hydroxyapatite (HAp, Ca_10_(PO_4_)_6_(OH)_2_) and β-tricalcium phosphate (β-TCP, Ca_3_(PO_4_)_2_) are the most commonly used.^3,4^ HAp is the most thermodynamically stable phase among CaPs in physiological environments and is the major mineral component of human bone, giving it the ability to bind directly to bone.^5,6^ In contrast, β-TCP is bioresorbable and can be replaced by new bone growth, which is a significant advantage over HAp.^5^ Due to its outstanding properties, β-TCP is used as a raw material for mineral bone cements, for bioceramic bone implant coatings as well as for dental applications.^1,7^ In addition to the mentioned factors, antibacterial properties are required in order to avoid the risk of infections after bone replacement surgery. Infections can lead to non-osseointegration and necrosis with serious consequences for patients.^3^ Furthermore, there is great concern about antibiotic resistance and the resulting mortality rate, which must also be considered.^3^ To address this serious problem, CaP can be doped with antibacterial metal ions, which was already successfully reported.^3,7,8^ In this context, Cu ions have shown to be promising candidates. They can easily penetrate bacterial membranes, mimic peroxidases and thus generate reactive oxygen species (ROS), which are able to destroy the bacterial structure.^3^ However, the optimal Cu concentration is still unknown, as it shows a dose-responsive effect and can be cytotoxic at higher concentrations.^3^ Therefore, control of Cu release is a crucial factor to destroy bacteria while minimizing harmful effects on normal eukaryotic cells. In addition, a controlled release of antibacterial metal ions can be achieved by the use of porous materials, where Cu ions are loaded into the CaP pores. In this context, porous TCP cements for controlled drug release have already been reported.^9^ Furthermore, porous CaP granules as well as ceramics have also been prepared to facilitate cell growth and infiltration.^6,10^ Higher porosity also provides a larger surface area, leading to increased resorption and cell-matrix interaction.^11^ An increase in porosity can also be achieved by the use of nanoparticles (NPs). Porous CaP NPs for drug delivery applications have already been reported in previous studies.^12–15^ Besides improved absorption by the body, mesoporous CaP NPs have a high surface-to-volume ratio and increased solubility.^16^ In order to stabilize NPs under physiological conditions, they can be processed into so-called supraparticles, which are typically micrometre-sized and composed of nanoparticular building blocks formed by self-assembly.^17^ As a result, the properties of primary NPs and microparticles can be combined, depending on the supraparticle structure.^18^ Supraparticle synthesis includes methods such as surface-templated evaporation, emulsion-templated self-assembly and microfluidic techniques.^19–22^ Apart from these processes, spray drying is a widely used and simple drying method in which an NP suspension is dried into a fine powder by spraying the feed into a hot drying medium.^17^ Liquid atomization leads to the evaporation of the solvent and the formation of supraparticles *via* droplet formation.^17^ The resulting supraparticle structure (*e.g.* raspberry-like, doughnut-like, dense or porous structure) can be easily adjusted by varying spray drying parameters such as primary NP concentration and size, drying temperature as well as use of particle mixtures.^17^ Supraparticles have already been reported in various applications, such as photonic devices, biosensing or catalysis.^18,23^ For biomedical applications, silica supraparticles have been used as drug delivery systems for the administration of neurotrophins or model proteins such as fluorescein isothiocyanate lysozyme.^23–25^ However, in the field of bone tissue regeneration, only a few studies have reported on the use of spray dried CaP particles, such as for additive manufacturing or as an implantable scaffold.^26,27^

Here, we present the preparation of spray dried Cu-doped CaP supraparticles with antibacterial properties for bone tissue regeneration. To this end, CaP NPs were prepared by a modified sol–gel process.^15^ To obtain the crystalline phase β-TCP, different Ca/P ratios (1.10–1.50) of the precursors were tested during wet-chemical step, followed by calcination of the NPs at 1000 °C.^28^ Subsequent, the NPs were processed into supraparticles by the spray drying method.^18^ To obtain an antibacterial effect, different Cu^2+^ concentrations (0.5–15.0 wt%) were also added in the process. In a second approach, the antibacterial properties of the uncalcined Cu-doped CaP supraparticles were tested against Gram-positive (*Bacillus subtilis* and *Staphylococcus aureus*) and Gram-negative (*Escherichia coli*) bacterial strains. For this purpose, the influence of different Cu amounts and its release were evaluated over 14 days. In addition, *in vitro* cytotoxicity and viability assays were performed using telomerase-immortalized human mesenchymal stromal cells (hMSC-TERT). hMSC-TERT were used as a model for primary bone marrow-derived stromal cells that have the ability to differentiate towards the osteogenic, adipogenic and chondrogenic lineages. However, primary cells are subject to the process of replicative senescence, which is why their cultivation time is limited. Overexpression of telomerase abrogates replicative senescence and hMSC-TERTs exhibit a high proliferation rate while maintaining their mesenchymal differentiation capacity.^29^

## 2 Materials and methods

### 2.1 Materials

Calcium nitrate tetrahydrate (Ca(NO_3_)_2_·4H_2_O, Sigma-Aldrich, ≥99%), ammonium phosphate dibasic ((NH_4_)_2_HPO_4_, Sigma-Aldrich, ≥99%), citric acid monohydrate (HOC(CO_2_H)(CH_2_CO_2_H)_2_·H_2_O, Sigma-Aldrich, 99.5–102%), cetyltrimethylammonium chloride solution (C_19_H_42_ClN, CTAC, Sigma-Aldrich, 25 wt% in H_2_O), ethylen glycol (HOCH_2_CH_2_OH, Carl Roth, ≥99%), triethanolamine (N(CH_2_CH_2_OH)_3_, thermo scientific, 99%), ethanolamine (NH_2_CH_2_CH_2_OH, Carl Roth, ≥99%), ethanol (C_2_H_5_OH, EtOH, Carl Roth, ≥99.5%), ammonium nitrate (NH_4_NO_3_, Carl Roth, ≥98%), copper nitrate trihydrate (Cu(NO_3_)_2_·3H_2_O, Merck, ≥99.5%), sodium hydroxide (NaOH, Carl Roth, ≥99%), Mueller Hinton Broth (MHB, Merlin Diagnostika), Minimum Essential Medium (MEM, Gibco), fetal calf serum (FCS, Bio & Sell), gentamicin sulfate and 100 nM sodium selenite (both Sigma-Aldrich), trypsin (Gibco), phosphate buffered saline (PBS, AppliChem GmbH), CellTiter-Glo Luminescent Cell Viability Assay (Promega GmbH), calcein AM and Hoechst (both Thermo Fisher Scientific), propidium iodide (Sigma-Aldrich), ethanol absolute p.A. (EtOH, AppliChem GmbH).

### 2.2 Synthesis of CaP NPs

CaP NPs were synthesized based on the modified Pechini sol–gel method described by Schirnding *et al.*^15^ Ca(NO_3_)_2_·4 H_2_O (1.50 g, 6.35 mmol) and citric acid monohydrate (1.20 g, 5.71 mmol) were dissolved in water (100 mL) in a 250 mL flask. (NH_4_)_2_HPO_4_ (762.50 mg/5.77 mmol; 699.0 mg/5.29 mmol; 645.22 mg/4.89 mmol; 599.11 mg/4.54 mmol; 559.0 mg/4.23 mmol) was added and stirred until everything was dissolved. This resulted in a molar ratio of Ca/P = 1.10–1.50. Then, the surfactant CTAC (3.21 mL) was introduced into the reaction and the mixture was stirred at 500 rpm. After 10 minutes (min), ethylene glycol (35.75 g; 0.58 mol) was added and the solution was cooled at 0 °C for 5 min. After the addition of triethanolamine (35.75 g; 0.24 mol) and ethanolamine (15.0 g; 0.25 mol), CaP NPs precipitated and stirring was continued for further 3 min. Subsequently, the NPs were separated by centrifugation (7129×*g*, 10 min) and redispersed in a mixture of NH_4_NO_3_/EtOH (2.0 wt%, 150 mL). To extract the surfactant, the particles were heated under reflux for 30 min, followed by centrifugation (7129×*g*, 10 min) and redispersion in ethanol (100 mL). After reheating under reflux conditions for further 30 min, the final NPs were obtained after centrifugation (7129×*g*, 10 min) and redispersion in water (50 mL). To obtain the crystal structure β-TCP, the CaP NPs were first dried in a vacuum oven (Memmert) at 90 °C and then calcined with a temperature rate of 2 °C min^−1^ at 1000 °C for 15 min (Nabertherm).

### 2.3 Preparation of Cu-doped CaP supraparticles

The preparation of Cu-doped CaP supraparticles was done by the spray drying method.^17^ For this purpose, a suspension of CaP NPs in water (4.4 wt%) was first prepared. Cu(NO_3_)_2_ (0.5–15.0 wt% based on CaP NPs) was added and stirred until a stable suspension was obtained. Spray drying was performed using a laboratory spray dryer (Büchi B-191) with an inlet temperature of 130 °C.^18^ The inlet hot air flow was set to 100% with a feed suspension flow of 15%.^18^ Crystalline structures were then obtained by calcining the supraparticles with a heating rate of 2 °C min^−1^ at 1000 °C for 15 min (Nabertherm). The procedure was repeated to produce CaP supraparticles without the use of Cu as a control.

### 2.4 Characterization methods of CaP particles

X-ray diffraction (XRD) of CaP particles was recorded using a diffractometer (D8 ADVANCE, Bruker) with Cu Kα radiation (*λ* = 1.5406 Å). A Si low background sample holder was used for the measurements. Scans were performed between 7° < 2*Θ* < 70° with an increment of 0.02° per step. The quantitative phase composition was determined by the profile fitting based software TOPAS 64, version 6. The hydrodynamic diameter of NPs in water was measured by using a dynamic laser scattering (DLS) instrument (Zetasizer Nano ZS, Malvern Panalytical). The reported values represent a mean of 3 measurements. Transmission electron microscopy (TEM) was used to characterize NP size and structure. Here, 10 μL of CaP NP suspension in water was placed on a 200-mesh carbon-coated copper grid (Sigma-Aldrich) and dried at room temperature (RT). TEM records were then obtained by a 200 kV analytical electron microscope (JEM-2100, JEOL). Approximately 30 NPs were measured manually and the average was calculated. The surface morphology and size distribution of CaP supraparticles were determined by scanning electron microscopy (SEM) measurements. The samples were prepared on a conductive carbon pad, followed by sputtering with platinum for 10 s at 30 mA and a distance of 9 cm (MED 010, Balzers Union). SEM images were obtained using an SE2 detector and an accelerating voltage of 1.5 kV (Supra 25, Zeiss). The Cu as well as the Ca and P amount in CaP particles were determined using inductively coupled plasma mass spectrometry (ICP-MS, iCAP RQ, Thermo Fisher Scientific). Prior to the measurements, each sample (3.0 mg) was dissolved in 69% nitric acid (1 mL; Carl Roth, ROTIPURAN® Supra 69%) for 24 hours (h), followed by dilution in extra pure water (1 : 100) with a conductivity of < 0.1 μS cm^−1^. 3 Measurements were performed per sample while the obtained concentrations were averaged afterwards. Cu, Ca and P contents were measured against standard solutions of 9.8 mg L^−1^ and 1000 mg L^−1^. The specific surface areas as well as pore volumes and diameters were measured by Brunauer–Emmett–Teller (BET) as well as by Barrett, Joyner and Halenda (BJH) methods using nitrogen adsorption at 77 K (Autosorb 3B, Quantachrome). The samples were degassed under vacuum at 110 °C for 16 h prior to the measurements.

### 2.5 Dissolution tests of Cu-doped CaP supraparticles

CaP supraparticles (0.10 g), doped with different Cu amounts (0.5–15.0 wt%) were incubated in bi-distilled water (1.0 mL) for 14 days at 37 °C and 200 rpm according to ISO standard 10 993-15 : 2019-11. Bi-distilled water was used to avoid possible interactions with salts or ions. The pH of the suspensions was adjusted to 7.4 ± 0.1 with 0.1 M NaOH. After 1, 2, 3, 7, 10 and 14 days, the supernatant (eluate) was removed by centrifugation (11 718×*g*, 15 min) and stored at 4 °C. The samples were again cultured with bi-distilled water and adjusted to a pH of 7.4 ± 0.1 during incubation. The Cu content was then determined using ICP-MS by diluting the samples (1.0 mL) in extra pure water (1 : 100). For the preparation of the antibacterial tests, the procedure was repeated for 24 h, where CaP supraparticles without Cu as a control were also tested.

### 2.6 Antibacterial testings of Cu-doped CaP supraparticles

For the determination of the antibacterial activity, the 24 h eluates of the Cu-doped CaP supraparticles (0–15.0 wt% Cu) were first sterilised with a 0.2 μL syringe filter. Then, the so-called inhibitor test was performed. It is a useful screening method to demonstrate the presence of inhibitors by inhibiting a reference pathogen (Bacillus subtilis–spore suspension (BGA/Merck 1.10649)). For this purpose, *B. subitilis* was evenly dissolved in MH agar at a defined number of bacteria and then poured into a Petri dish and waited until the agar solidified. One drop (10 μL) of each eluate was then placed on the agar plate. If the fluid has an inhibitory effect, an inhibition zone (ZOI) forms around the drop after incubation at 36 °C for 24 h.^30^

The bacterial strains *Staphylococcus aureus* (ATCC 29213) and *Escherichia coli* (ATCC 25922) were used to determine the inhibitory effect of the eluates. For this purpose, the test organisms were adjusted to 0.5 McFarland (≈1.5 × 10^8^/KBE per mL) with physiological NaCl solution and diluted with MHB 1 : 10. 5 μL of this dilution was then pipetted into wells containing the 24 h Cu eluates (100 μL).^31^ This corresponds to an approximate bacterial count of 5 × 10^5^/KBE per mL (EN ISO 20776-1 and EUCAST).^32^ In addition to the 24 h CaP eluate without Cu, a bacterial solution (100 μL) without eluates was also applied as a growth control (GC) and blank values (only MHB) in microtiter plates without bacteria. To test the antibacterial effect of Cu alone, Cu(NO_3_)_2_·3H_2_O in water was also used at various concentrations (1–50 mg L^−1^). The samples were then incubated at 36 °C for 20 h. Bacterial growth was identified when the samples became turbid. In order to determine whether the Cu eluates had a bactericidal effect on the test organisms, 10 μL of the wells with no visible growth (5.0–15.0 wt% Cu) as well as of the tested Cu(NO_3_)_2_ solutions were plated on a Columbia blood agar plate (Thermo Fisher Scientific). The solutions were incubated for 24 h at 36 °C and the bactericidal effect was determined.^31^ All tests were performed in triplicate to confirm the activity.

### 2.7 *In vitro* cytocompatibility studies of Cu-doped CaP supraparticles

#### 2.7.1 Cell culture

hMSC-TERTs, which show high proliferation rate while maintaining their mesenchymal differentiation capacity *in vitro* and *in vivo*, were established by M. Kassem's group (Odense, Denmark).^33,34^ Cells were cultured in Minimum Essential Medium (MEM) with 10% (v/v) fetal calf serum (FCS), 50 μg mL^−1^ gentamicin sulfate, and 100 nM sodium selenite at 37 °C in a 95% humidified air and 5% CO_2_ atmosphere.

#### 2.7.2 Sterilization of supraparticles

Prior to use, the supraparticles underwent three rounds of EtOH washing to ensure sterility. Subsequently, they were washed 3 times with medium and stored at 4 °C.

#### 2.7.3 Cell viability assay

To examine the cell viability, hMSC-TERT were seeded in a 96-well plate (1 × 10^3^ cells per well) at 37 °C and 5% CO_2_ overnight for adherence. Cells were treated with the Cu-doped (0.5–15.0 wt%) and undoped CaP supraparticles at 4 concentrations (10 mg mL^−1^, 1 mg mL^−1^, 0.1 mg mL^−1^, 0.01 mg mL^−1^) and incubated for 24, 48, or 72 h. Viability rates were assessed using the CellTiter-Glo Luminescent Cell Viability Assay according to the manufacturer's instructions. Luminescence was measured using an Orion II luminometer (Berthold Detection Systems). Each reaction was performed in triplicate and the luminescence values were averaged. 3 independent experiments were conducted and each reaction was performed in triplicate. Luminescence values of the individual experiments were averaged, normalized to the respective control and the overall mean was calculated.

#### 2.7.4 Live/dead assay

For the evaluation of the ratio of viable cells to dead cells, hMSC-TERT were seeded in a 96-well plate (1,5 × 10^4^ cells per well) at 37 °C and 5% CO_2_ overnight for adherence. Cells were then stimulated with the Cu-doped (0.5–15.0 wt%) and undoped CaP supraparticles at concentrations of 10 mg mL^−1^, 1 mg mL^−1^, 0.1 mg mL^−1^, and 0.01 mg mL^−1^ and incubated for 24, 48, or 72 h. 100 μM calcein AM and 200 μM propidium iodide were dissolved in PBS. A mixture of both solutions was then prepared for live/dead staining and Hoechst solution (2.5 μg mL^−1^) was added. 150 μL of the staining solution was dispensed into each well. The cells were then incubated for 15 min at RT and examined using a Zeiss Axio Observer 7 microscope (ZEISS) with a 10× objective. 3 independent experiments were performed and nine photos of each sample were taken. Analysis was facilitated using ZEISS ZEN 2.6 Pro software, which incorporates the ZEN Intellesis module designed for segmenting and quantifying live and dead cell signals.

## 3 Results and discussion

### 3.1 Synthesis and characterization of CaP NPs

**Fig. 1:**
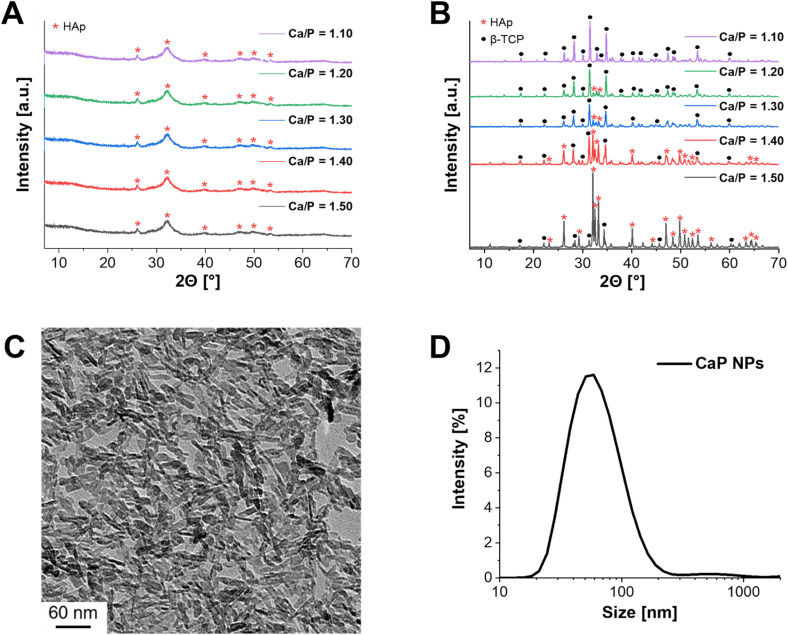
Characterization of CaP NPs. (A) XRD pattern of uncalcined CaP NPs with different Ca/P molar ratios (1.50–1.10). (B) XRD pattern of calcined CaP NPs with different Ca/P molar ratios (1.50–1.10). (C) TEM images of uncalcined, nanorod-shaped CaP NPs (Ca/P = 1.10) with a size of 28 ± 15 nm in length and 6 ± 4 nm in width. (D) Hydrodynamic diameter of uncalcined CaP NPs in water (Ca/P = 1.10): 68 ± 35 nm.

In the first step of the synthesis, amorphous CaP NPs were precipitated, followed by calcination to form crystalline structures (Fig. 1A and B). In order to obtain the desired crystalline phase β-TCP, the Ca/P molar ratio was set to 1.50 during synthesis.^16^ XRD measurements were performed to verify the crystalline structure of the calcined CaP NPs. In this context, powder diffraction was first measured for the uncalcined CaP NPs (Fig. 1A – Ca/P = 1.50). As a result, low crystalline HAp was detected. This is in agreement with the results of Schirnding *et al.* showing that amorphous CaP crystallizes over time in aqueous solution.^15^ In contrast, calcination at 1000 °C for 15 min resulted in the crystalline phase of HAp (≈90%) with only a small share of β-TCP (≈10%) (Fig. 1B – Ca/P = 1.50). To check whether the purification steps by centrifugation during the synthesis have an influence on the crystal structure of CaP, the unpurified NPs were also calcined and the crystal structure determined. In fact, the crystal structure differs from that of the purified calcined NPs and resulted in the complete formation of β-TCP (Fig. S1, ESI†). The reason for the different crystal structures can be explained by the incomplete precipitation of the NPs. Only part of the PO_4_^3−^ can react with Ca^2+^, resulting in a higher molar Ca/P ratio. Through the following purification steps, the remaining PO_4_^3−^ ions were removed from the solution. ICP-MS was applied to measure the experimental Ca/P ratio. In fact, a ratio of 1.67 ± 0.01 was obtained, corresponding to the theoretical ratio of HAp.^16^

In the next step, the Ca/P molar ratio was gradually reduced (Ca/P = 1.50–1.10) and the synthesized NPs were calcined, to check the effect on the β-TCP share. While the crystal structure of uncalcined CaP NPs did not change with decreasing ratio (Fig. 1A), calcination, on the other hand, showed an increase in the formation of β-TCP as well as a decrease in HAp formation (Fig. 1B). A Ca/P ratio of 1.10 resulted in a single crystalline phase of β-TCP. This was emphasized by ICP-MS with Ca/P = 1.55 ± 0.03, which is close to the theoretical value of β-TCP (1.50).^16^ Therefore, further characterizations were performed only for the CaP NPs with Ca/P = 1.10. The size and structure of the resulting NPs were characterized using DLS and TEM. DLS measurements provided a hydrodynamic diameter of 68 ± 35 nm in water (Fig. 1D) while TEM images showed that the NPs had a nanorod-like structure with a size of 28 ± 15 nm in length and 6 ± 4 nm in width (Fig. 1C).

During the synthesis the surfactant CTAC was added to achieve a mesoporous structure of the CaP NPs.^15^ To verify the mesoporosity, N_2_ adsorption measurements were performed. The BET surface area was 105 m^2^ g^−1^ with a pore diameter distribution of 1–14 nm and a pore volume of 0.27 cm^3^ g^−1^. Typically, microporous NPs have a pore size of less than 2 nm, while mesopores are between 2 and 50 nm.^35^ Therefore, a mixture of micro- and mesopores was present in the synthesized CaP NPs. However, the specific surface area and pore volume were significantly smaller compared to the NPs described by Schirnding *et al.* (900 m^2^ g^−1^ and 1.0 cm^3^ g^−1^).^15^ In addition, the NP shape differed from the previous study.

While Schirnding *et al.* showed a porous spherical structure in the SEM images, the described CaP NPs in this study have a rod-like shape (Fig. 1C).^15^ Since only the Ca/P ratio was varied (increase of Ca(NO_3_)_2_ amount) and (NH_4_)_2_HPO_4_ instead of ammonium dihydrogenphosphate ((NH_4_)H_2_PO_4_) as PO_4_^3−^ salt was used in contrast to Schirnding *et al.*, this may be the reason for the difference in structure and porosity. To understand the discrepancy in more detail, it is necessary to adjust other parameters of the synthesis, *i.e.* to increase the amount of the surfactant CTAC.

### 3.2 Synthesis and characterization of Cu-doped CaP supraparticles

**Fig. 2:**
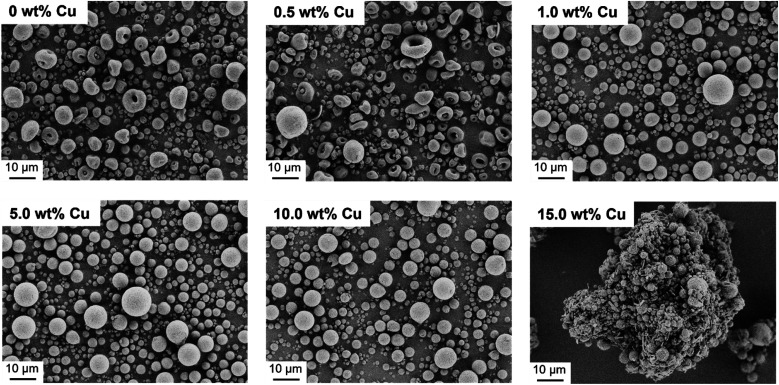
Spray dried uncalcined Cu-doped CaP supraparticles (Ca/P = 1.10) without Cu (0 wt% Cu) and with different Cu-concentrations (0.5–15.0 wt%). The particle morphology changed from mushroom- and doughnut-like (0 and 0.5 wt% Cu) to spherical particles (1.0–10.0 wt%) with the increasing of Cu concentration. Further increasing of the Cu amount resulted into particle agglomeration (15.0 wt%).

After successful synthesis and characterization, CaP NPs with a molar ratio of Ca/P = 1.10 were processed into supraparticles by the spray drying method.^18^ A part of the CaP NPs was also doped with different Cu concentrations (0.5–15.0 wt%) to achieve an antibacterial effect. In this context, Cu^2+^ in the form of Cu(NO_3_)_2_ was added to the NP-water suspension (4.4 wt%) prior to spray drying. The yield of the spray drying was approximately 60% due to particle loss in the glass apparatus. The spray dried CaP without and with Cu^2+^ are shown in Fig. 2. The CaP supraparticles without Cu^2+^ and with only a low Cu concentration (0.5 wt%) show mushroom- and doughnut-shaped structures with a size of 1–18 μm (Fig. 2). This phenomenon is usually observed in the case of stable suspensions with small NPs.^18^ During the spray drying process, the NPs accumulate on the outside of the droplet due to small van der Waals and capillary forces, trapping the water inside the droplet.^18,36,37^ The subsequent evaporation of the solvent leads to a deformation of the supraparticle structure, resulting in doughnut- or mushroom-shaped particles. On the other hand, increasing the Cu concentration (1.0–15.0 wt%) led to the formation of spherical structures with a size of 1–12 μm due to destabilization and agglomeration of the NPs dispersion caused by the Cu(NO_3_)_2_.^38^ In addition, the use of high Cu amount (15.0 wt%) led to agglomeration of the supraparticles and formation of a Cu(NO_3_)_2_ layer around the supraparticles (Fig. 2).

**Fig. 3:**
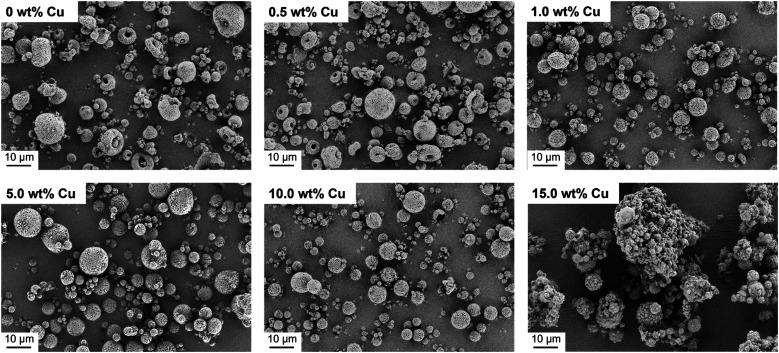
SEM images of calcined Cu-doped CaP supraparticles (0–15.0 wt% Cu). Calcination led to no destruction of supraparticle shape but to aggregation of supraparticles and melting of primary NPs.

In the next step, the spray dried Cu-doped CaP supraparticles (0–15.0 wt%) were calcined at 1000 °C to obtain the desired crystal phase β-TCP. In this context, calcination did not lead to any destruction of the supraparticle shape (Fig. 3). However, the primary NPs melted together within the supraparticle and the supraparticle surface became holey due to the thermal decomposition of organic substances such as ethylene glycol or citric acid during calcination.^39^ In addition, aggregation between the primary NPs and the supraparticles was observed, which is a typical feature of the calcination process.^40,41^

**Fig. 4:**
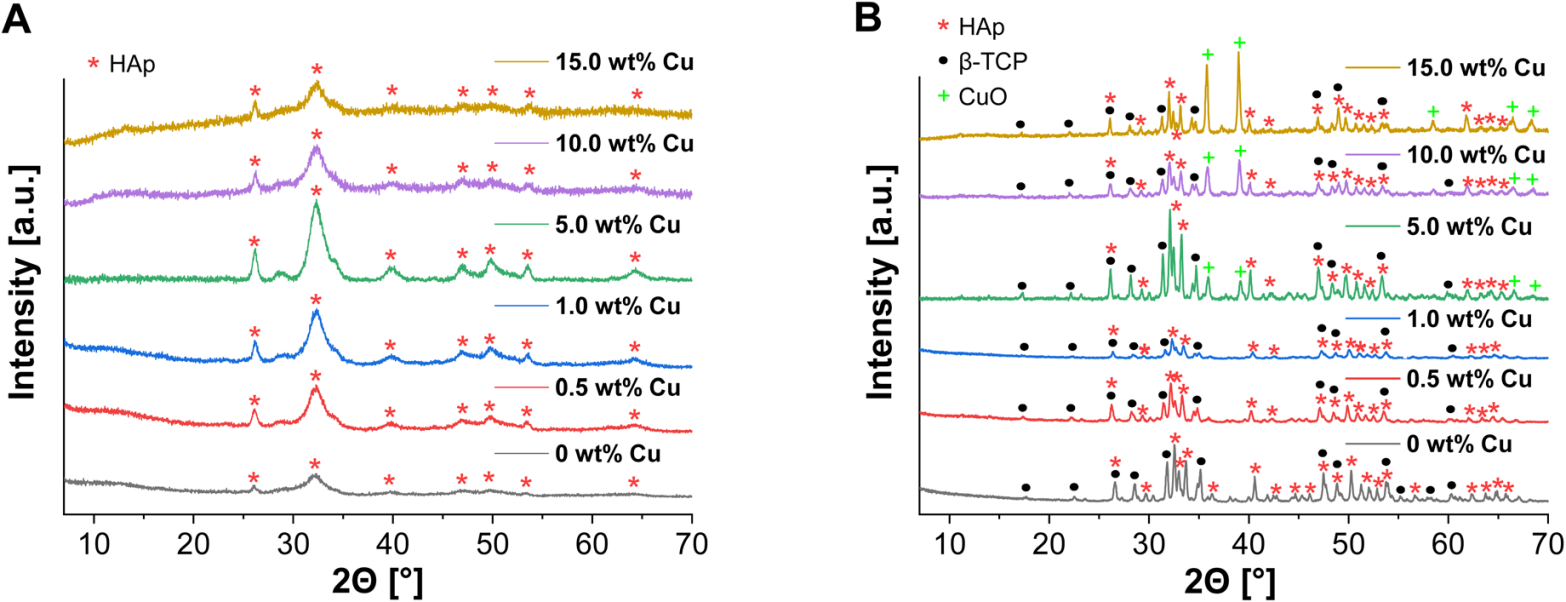
XRD pattern of spray dried uncalcined (A) and calcined (B) Cu-doped CaP supraparticles (0–15.0 wt% Cu).

ICP-MS measurements were performed, to confirm the incorporation of Cu^2+^ into the supraparticles as well as to check the influence of the Cu^2+^ amount on the spray drying and calcination process. As a result, Cu^2+^ could be detected with no significant changes in concentration, but calcination of the supraparticles resulted in an increase in the relative Cu^2+^ concentration depending on the amount of Cu used (Table 1). This can also be explained by mass losses (≈20–50%) during calcination due to thermal decomposition of the organic additives.^39^ Similar mass losses (≈40%) were also observed in earlier work by Wehl *et al.* who prepared mesoporous magnesium phosphate (MgP) NPs by the Pechini sol–gel process and determined the mass changes of the NPs up to 850 °C by thermogravimetric analysis.^42^ Besides ICP-MS, XRD was measured to analyze the incorporation of the Cu^2+^ into the crystal structure and the formation of β-TCP after calcination. In relation to all uncalcined Cu-doped supraparticles, no diffraction peaks of Cu(NO_3_)_2_ were detected (Fig. 4A) and there were no significant differences compared to the XRD pattern of the primary CaP NPs (Fig. 1A). The incorporation of Cu^2+^ into the crystal structure could not be demonstrated, which means that Cu had no effect on the crystal structure. On the other hand, the XRD pattern of calcined Cu-doped CaP supraparticles showed the presence of CuO (tenorite), especially for the particles with 5.0–15.0 wt% Cu (Fig. 4B). CuO typically forms during calcination in oxygen atmosphere.^43,44^ The decomposition of Cu(NO_3_)_2_ to CuO also explains the mass losses as well as the increase in the relative Cu^2+^ concentration during calcination (Table 1). With higher Cu content, the CuO peak in XRD images increased. In addition, calcination led to crystalline phases of HAp (≈70%) and β-TCP (≈30%) although a Ca/P molar ratio of 1.10 was used. In this context, an experimental Ca/P molar ratio of 1.79 for CaP supraparticles was detected by ICP-MS. During spray drying, rapid solvent evaporation and mechanical atomization can generate localized high temperatures and stresses, leading to the potential loss or alteration of unstable surface PO_4_^3−^ ions. This resulted in a higher Ca/P ratio.

**Table 1:** Cu amounts of calcined CaP supraparticles (0.5–15.0 wt% Cu) experimentally determined by ICP-MS

Theoretical Cu amount [wt%]	0.5	1.0	5.0	10.0	15.0
Experimental Cu amount [wt%]	0.6	1.2	6.9 ± 0.2	13.3 ± 0.5	18.0 ± 1.0

Nevertheless, previous studies have shown that biphasic CaP has advantages over monophasic CaP.^8,45^ For example, the combination of poorly soluble HAp with highly resorbable β-TCP resulted in a better control of bioactivity and degradation.^45^

In addition, N_2_ adsorption measurements were carried out to check whether the formation of supraparticles and their subsequent calcination had an effect on the porosity compared to the primary NPs. In the case of the uncalcined supraparticles, no significant changes were found, compared to the primary CaP NPs. In contrast, a drastic loss of porosity was observed for the calcined Cu-doped CaP supraparticles. The BET surface area decreased to 3 m^2^ g^−1^ with a pore size of 2–4 nm and with no significant pore volume (0.0059 cm^3^ g^−1^). Therefore, the loss of mesoporosity of the supraparticles was a consequence of the calcination process that led to NP melting and aggregation. The uncalcined CaP supraparticles were initially selected for the following antibacterial and cellular tests because they were easy to produce without changing the Cu concentration after spray drying. In contrast, calcination resulted not only in a change of Cu concentration but also in a loss of porosity and mass.

### 3.3 Dissolutions tests of Cu-doped CaP supraparticles

**Fig. 5:**
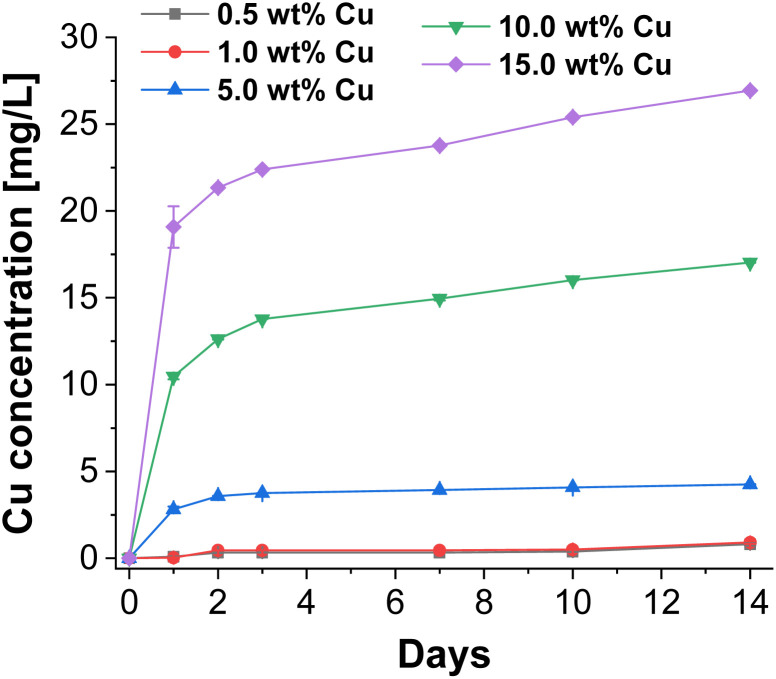
Released cumulative Cu concentrations of CaP supraparticles using different Cu concentrations (0.5–15.0 wt%) in bi-distilled water over a period of 14 days.

To investigate the antibacterial potential of the prepared particles, Cu release experiments of CaP supraparticles with different Cu amounts (0.5–15.0 wt%) in bi-distilled water were performed over a period of 14 days. In this process, the supernatant (eluate) was removed at different time points and its Cu content was determined by ICP-MS. The results are shown in Fig. 5. When using a Cu concentration of 0.5–1.0 wt%, almost no release was observed over the whole period compared to the samples with higher Cu concentrations (≥5.0 wt%). The release after 14 days was 0.8 mg L^−1^ for the eluate with 0.5 wt% Cu and 0.9 mg L^−1^ using a Cu concentration of 1.0 wt%. On the other hand, the eluate with 5.0 wt% Cu, showed a slightly higher release, but also increased slowly over the entire period (final concentration = 4.2 mg L^−1^). In comparison, the 10.0 wt% and 15.0 wt% Cu eluates had significantly higher releases with a stronger increase over the examined period of 14 days. The final concentration was 17.0 mg L^−1^ for the 10.0 wt% Cu eluate and 26.9 mg L^−1^ for the 15.0 wt% Cu eluate. Overall, all eluates showed a sustained slow release over the entire period. Depending on the Cu concentration, the total release was between 0.08–0.19% relative to the Cu amount in the supraparticles. This release is desirable for bone regeneration applications where an immediate but slow sustained release of Cu is required to prevent biofilm formation and therefore infection. For example, in large bone defects or chronic bone diseases where the bone cannot heal itself, bone regeneration is slow and requires significant support. In addition, bone implants are often associated with infections, which could be inhibited by applying such an antibacterial coating.

In general, there was a concentration-dependent increase in Cu release for the different Cu-doped CaP supraparticles. In addition, not only the Cu concentration but also the porosity and solubility of the supraparticles played a role in the amount of release. The Cu release can therefore also be explained by the porosity of the supraparticles, which allowed Cu to diffuse out of the pores of the supraparticles. In terms of the crystal structure, particles were composed of HAp which has a lower solubility compared to other CaP crystal structures such as β-TCP.^46^ However, the present supraparticles had only a low degree of crystallinity, which means that the solubility should be even higher compared to highly crystalline HAp.^47^

For this reason, in addition to Cu release, Ca and P release was also determined over 14 days. The results are shown in Fig. S2 ESI.† This demonstrates that there was already a significant release of Ca and P ions during this period. However, Ca ion release was much higher than P ion release depending on Cu content. This may be due to the fact that Ca ions are likely to be localized on the surface of the particles or in poorly crystalline areas which dissolve more rapidly. The low crystallinity of the CaP particles favored this. The Ca/P ratio also played a role. As the Ca content was higher than the P content (Ca/P = 1.79), more Ca ions could be released than P ions. The release is also influenced by the Cu content. As the Cu content increased, more Ca ions but less P ions were released. This can be explained by the fact that Cu could interact more strongly with P and form stable complexes which could inhibit the release of P ions.^48^

In conclusion, it can be shown that in addition to porosity, the dissolution of the particles must also have a decisive influence on Cu release. However, these two factors were similar for all tested particles and should only play a minor role when comparing the different supraparticles.

### 3.4 Antibacterial testings of Cu-doped CaP supraparticles

For antibacterial testing, 24 h eluates were selected to provide a direct antibacterial effect for later use, which should prevent biofilm formation and thus infection. The antimicrobial activity of the eluates from the Cu-doped CaP supraparticles (0–15.0 wt% Cu) was first tested with the Gram-positive standard bacterium *B. subtilis*. For this purpose, the so-called inhibitor test was used, where the sample can be identified as antimicrobial if the ZOI is ≥ 2 mm.^49^ The results are stated in Table 2 and Fig. S3 ESI.† For Cu concentrations <5.0 wt%, no antimicrobial activity (ZOI) was observed after 24 h incubation. In contrast, ZOIs were observed for 5.0–15.0 wt% Cu eluates (Fig. S3 ESI†), with increasing ZOIs from 5.0 (ZOI = 14 mm) to 10.0 wt% Cu (ZOI = 17 mm). In contrast, for the 15.0 wt% Cu eluate, the ZOI decreased again (9 mm).

**Table 2:** Results of antibacterial testings with eluates from Cu-doped CaP supraparticles (0–15.0 wt% Cu)

CaP eluate	*S. aureus*		*E. coli*		*B. subtilis*
	Growth inhibition	Bactericidal effect	Growth inhibition	Bactericidal effect	ZOI
Eluate without Cu	0%	0%	0%	0%	0 mm
0.5 wt% Cu eluate	0%	0%	0%	0%	0 mm
1.0 wt% Cu eluate	0%	0%	0%	0%	0 mm
5.0 wt% Cu eluate	100%	100%	100%	100%	14 mm
10.0 wt% Cu eluate	100%	100%	100%	33.3%	17 mm
15.0 wt% Cu eluate	100%	66.6%	100%	100%	9 mm

In addition to *B. subtilis*, the Gram-positive bacterium *S. aureus* and the Gram-negative bacterium *E. coli* were also tested as they are among the most common pathogens present after orthopaedic surgery.^50,51^ After 20 h of incubation with the 24 h eluates, there was no sign of bacterial growth for *S. aureus* and *E. coli* in the eluates containing 5.0–15.0 wt% Cu, indicating that these concentrations are growth inhibitory (Table 2). In contrast, *E. coli* and *S. aureus* were able to grow in the eluate with Cu concentrations of 0.5 and 1.0 wt%. The CaP control without Cu as well as the GC also showed significant growth of *S. aureus* and *E. coli*, confirming the results of the experiments. To determine the bactericidal effect on the test organisms, the Cu eluates with no visible growth (5.0–15.0 wt%) were again incubated for 24 h. No growth of *E. coli* and *S. aureus* was observed for the eluates with 5.0 wt% Cu, representing 100% growth inhibition (Table 2). In addition, *E. coli* didn't grow on samples with a Cu concentration of 15.0 wt% while in contrast the 10.0 wt% Cu eluate showed no bacterial growth in only one (33.3%) out of three cases, as indicated by the visible turbidity. In the case of *S. aureus* testings, the eluate containing 10.0 wt% Cu exhibited no bacterial growth standing in contrast to the 15.0 wt% Cu sample. Here two testings (66.6%) did not exhibit any existence of bacteria but nevertheless in one case it was shown that the inhibition of bacterial growth was not sufficient due to the turbidity.

In this study, complete antibacterial activity was demonstrated only for the eluate with a Cu concentration of 5.0 wt%. Below 5.0 wt% Cu, the eluates showed no bacterial inhibition. The 10.0 wt% and 15.0 wt% Cu eluates showed growth inhibition of *S. aureus* and *E. coli*, but only a limited bactericidal effect after re-incubation for 24 h. Since the Cu release increased with higher Cu concentration, the samples with increasing Cu concentration should have a higher antibacterial activity, which was not the case in this study. It was noticed that the 5.0–15.0 wt% Cu eluates formed a white precipitate when stored at 4 °C, which was unstable and disappeared with increasing temperature (RT).

In order to analyze these precipitates, the 5.0–15.0 wt% Cu eluates were additionally freeze-dried (Christ, alpha 2-4 LSCplus) and the crystal structure was determined by XRD. The precipitate was composed of NaNO_3_, which is shown in Fig. S4 ESI† as an example for the 10.0% Cu eluate. The formation of precipitate can be explained by the neutralization of the supraparticle suspensions with NaOH and the used Cu(NO_3_)_2_ which are able to react and thereby causes precipitation of NaNO_3_. In general, NaNO_3_ has no bacteriostatic effect, but it can be reduced to NaNO_2_ by bacterial nitratase.^52^ NaNO_2_ is commonly used in the food industry as a preservative to prevent biological spoilage.^53^ It is believed that the reduction of NO_3_^−^ to NO_2_^−^ results in the formation of hydroxylamine, which inactivates catalase and accumulates hydrogen peroxide.^52^ This is toxic to bacteria even at very low concentrations. The inhibition is also pH dependent, with some studies showing a stronger effect in acidic conditions.^52,54,55^ Nevertheless, to determine the antibacterial effect (growth inhibition and bactericidal effect) of Cu in Cu(NO_3_)_2_, pure Cu(NO_3_)_2_ was also tested at different concentrations (1–50 mg L^−1^) in bi-distilled water on *E. coli* and *S. aureus*. As a result, Cu(NO_3_)_2_ was found to have an antibacterial effect on *E. coli* only at a concentration of ≥20 mg L^−1^ (≙ 5.6 mg per L Cu^2+^), while the minimum inhibitory concentration (MIC) for *S. aureus* was even higher at 50 mg L^−1^ (≙ 13.1 mg per L Cu^2+^).

Overall, the antibacterial Cu(NO_3_)_2_ concentration for *E. coli* was about 2 times higher than for the 5.0 wt% Cu eluate. In contrast, the MIC for *S. aureus* was already more than 4 times higher than in the 5.0 wt% Cu eluate. This confirmed that the precipitation of NaNO_3_ must have a decisive influence on the antibacterial effect. The amount of NO_3_^−^ released and probably reduced to NO_2_^−^ in each eluate needs to be analyzed in further studies. However, as the 5.0 wt% Cu eluate showed complete antibacterial inhibition in all tests performed, it is assumed that the effect of NO_2_^−^ is strongest in this case.

In general, the antibacterial activity of NaNO_2_ also depends on many factors, such as the bacterial organism and the pH.^55^ For example, in the literature the MIC for *E. coli* at pH 6.3 is between 100 and 200 μmol mL^−1^, while at pH 5.4 it is slightly lower at >10 μmol mL^−1^.^54,55^ In contrast, the MIC of Cu in this study is 0.09 μmol mL^−1^ (5.6 mg L^−1^) for *E. coli*, which is significantly lower than the MIC of NO_3_^−^. Therefore, it is believed that the antibacterial activity of Cu should be higher than that of NO_3_^−^, but this also needs to be analyzed in further work.

With regard to Cu, the antibacterial activity also depends on its used form (NP, salt, Cu valency).^56^ For example, CuO NPs showed a minimum inhibitory concentration (MIC) of 2.50 mg mL^−1^ against *E. coli* and *S. aureus*.^57^ In contrast, the MIC for CuSO_4_ was between 100 and 200 μg mL^−1^, depending on the bacterium used.^58^ However, research on the MIC of Cu(NO_3_)_2_, which was used in that study, was limited. One study showed that the lower detection limit was reached after 60 min incubation with *E. coli* and 300 min incubation with *S. aureus* using a Cu(NO_3_)_2_ concentration of 24.2 mg mL^−1^.^59^ This value is significantly lower when compared to the MIC of *E. coli* and *S. aureus* found in the present study (20 and 50 mg L^−1^).

Therefore, the specific bacterial organism also plays an important role in the antibacterial activity. For example, it has already been reported that *S. aureus* is less sensitive to Cu NPs than the Gram-negative bacterium *E. coli*, which was also observed in the present study.^56^

### 3.5 *In vitro* cytocompatibility studies of Cu-doped CaP supraparticles

**Fig. 6:**
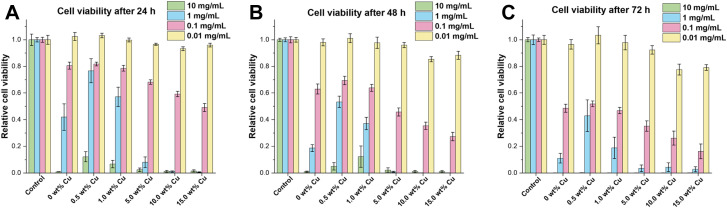
The cell viability of hMSC-TERT cells was evaluated after exposure to Cu-doped (0.5–15.0 wt%) and Cu-undoped CaP supraparticles at concentrations of 10, 1, 0.1 and 0.01 mg mL^−1^ for (A) 24 h, (B) 48 h and (C) 72 h. The control group consisted of untreated hMSC TERTs. Data points represent the normalized mean value of ATP present in culture ± SEM (*n* = 3) performed in triplicates by CellTiter-Glo® viability assay. Statistical analysis was performed by 2way ANOVA *via* GraphPad Prism. Data was compared with the control (*: *p* < 0.05; **: *p* < 0.01; ***: *p* < 0.001; ****: *p* < 0.0001).

To study the effect of the uncalcined Cu-doped CaP supraparticles on hMSC-TERTs, the amount of ATP in culture was measured as an indicator of metabolically active cells. Therefore, cells were stimulated with the supraparticles at concentrations ranging from 0.01 to 10 mg mL^−1^ and the cell viability was measured at 24, 48 and 72 h. All supraparticles exhibited cytotoxic effects on hMSC-TERTs when treated with a concentration of 10 mg mL^−1^ at 24, 48, and 72 h (Fig. 6). At a concentration of 1 mg mL^−1^, the cells stimulated with the undoped and Cu-doped (0.5–1.0 wt%) supraparticles showed lower metabolic activity compared to the control (untreated cells). In contrast, the hMSC-TERTs stimulated with the Cu-doped supraparticles containing 5.0–15.0 wt% Cu exhibited hardly any measurable metabolic activity. In contrast, when hMSC-TERTs were exposed to the supraparticles at a concentration of 0.1 mg mL^−1^ for 24, 48, and 72 h, a clear tendency was observed. The amount of ATP measured decreased with increasing Cu weight fraction. In comparison, a concentration of 0.01 mg mL^−1^ did not exhibit any cytotoxic effects on the cells after 24 h (Fig. 6A). These findings support the rough estimation that CaP concentrations below 0.1 mg mL^−1^ are not harmful in cell culture.^60^ According to the literature, CaP NPs are not inherently cytotoxic, but the release of Ca ions in high concentration after dissolution can be damaging to a cell.^60^ In addition, non-functionalized NPs often exhibit agglomeration in solution, leading to strong sedimentation of the NPs on cells. This results in high intracellular CaP concentrations and uptake, followed by cell death.^60^ The cytotoxicity of CaP particles using concentrations ≥0.1 mg mL^−1^ was therefore also confirmed in this study. After 72 h of exposure, cells treated with CaP supraparticles containing more than 5.0 wt% Cu showed a significant decrease in ATP levels compared to the control group (Fig. 6C; *p* ≤ 0.008). The results indicate that the cytotoxic effect on cells was enhanced with an increase in the Cu content of the supraparticles. The toxicity of Cu to essential biomolecules was also been reported in numerous studies.^3,61,62^ Another observation was that an increase in particle incubation time was accompanied by a corresponding decrease in cell viability. For example, the supraparticles with 15.0 wt% Cu at a concentration of 0.1 mg mL^−1^ showed a reduction in cell viability of about 50% after 24 h compared to the control. The values decreased to 30% after 48 h and to only 20% after 72 h.

In conclusion the stimulation with 0.01 mg mL^−1^ appeared to have a minimal, if any, adverse effect on cell viability. However, there was a slight negative effect of supraparticles with a Cu content exceeding 5.0 wt% after 72 h. At a concentration of 0.1 mg mL^−1^, the supraparticles had a time- and Cu-dependent inhibitory effect on cell viability.

In contrast, for cells treated with supraparticle concentrations of 1 or 10 mg mL^−1^, no or only low metabolic activity after 24 h was shown.

**Fig. 7:**
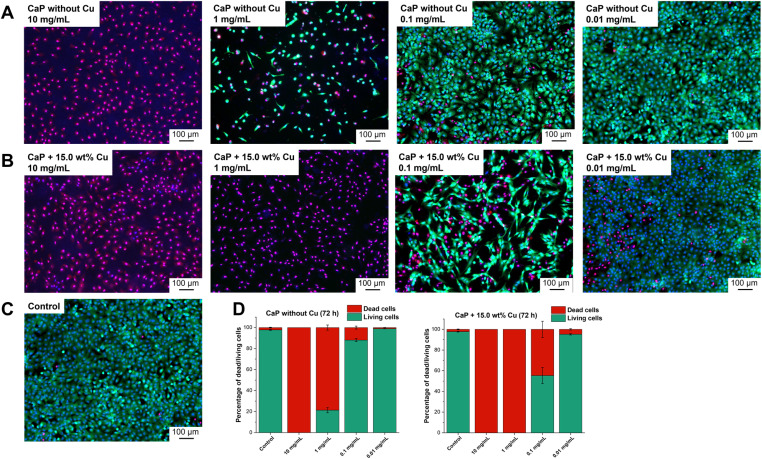
Live/dead staining 72 h after incubation with CaP supraparticles without (A) and with 15.0 wt% Cu (B) as well as of the control (C). Green: Fluorescence signal of living cells. Magenta: Fluorescence signal of dead cells. Blue: Fluorescence of cell nucleus. Representative images of three independent experiments are shown. (D) Percentage of dead/living cells of live/dead staining 72 h after incubation with undoped and Cu-doped (15.0 wt%) CaP supraparticles. The diagrams display the results of quantitative analysis of fluorescence micrographs from 3 independent experiments and 9 photos of each sample ± SEM. The analysis was performed using ZEISS ZEN 2.6 Pro software and the ZEN Intellesis module.

The investigation of cell viability was supported by live/dead staining, which allows the distinction between living and dead cells. Propidium iodide identifies dead cells while Calcein-AM stains living cells. The intensity of the staining is represented by light to dark green colour in the shown images (Fig. 7). The effects of CaP supraparticles without Cu (Fig. 7A) and with 15.0 wt% Cu (Fig. 7B) as well as of the control (Fig. 7C, untreated cells) on hMSC-TERTs after 24, 48 and 72 h were compared. Fig. 7 shows the results after 72 h. Data for 24 and 48 h are shown in the supplement (Fig. S5 and S6 ESI†). It was observed that a concentration of 10 mg mL^−1^ had a toxic effect on the hMSC-TERTs, resulting in a culture consisting of exclusively dead cells. A clear difference in the effect of the two supraparticles was observed at a concentration of 1 mg mL^−1^. Living cells were detected in the culture containing undoped CaP supraparticles, while only dead cells were found in the culture containing Cu-doped supraparticles. When stimulated with a concentration of 0.1 mg mL^−1^, the cultures consisted mainly of living cells. The number of dead cells varied between the two types of supraparticles. In the absence of Cu, the CaP supraparticles exhibited a dead cell frequency of 11.9% dead cells, whereas the CaP supraparticles containing 15.0 wt% Cu showed a higher incidence of 44.7% dead cells (Fig. 7D). The live/dead staining revealed that most of the cells were alive after treatment with the particles at a concentration of 0.01 mg mL^−1^, and very few dead cells were observed. Overall, the results of the live/dead images are consistent with the viability measurements (Fig. 6).

## 4 Conclusions

The aim of this study was to prepare Cu-doped CaP supraparticles with high antibacterial activity to improve bone tissue regeneration. CaP NPs were successfully prepared using a modified sol–gel process followed by spray drying with Cu^2+^ to form supraparticles. Complete bacterial inhibition was achieved for all tested bacterial strains (*B. subtilis*, *S. aureus* and *E. coli*) using uncalcined CaP supraparticles with a Cu concentration of 5.0 wt%. In contrast, higher Cu concentration (10.0 and 15.0 wt%) showed only a limited bactericidal effect after re-incubation for 24 h. The formation of a NaNO_3_ precipitate and its reduction to the antibacterial NaNO_2_ may be the reason for the differences in antibacterial activity. The actual influence of NO_3_^−^ and its assumed reduction to NO_2_^−^ still needs to be investigated in the future. In this context, NaNO_3_ could be tested directly to see if it exhibits antibacterial activity even without Cu. In order to determine the antibacterial effect of the added Cu alone in the CaP supraparticles, future tests would also need to use a different Cu salt than the Cu(NO_3_)_2_. For example, the calcination of Cu-doped CaP supraparticles resulted in the formation of CuO, whose antibacterial effect could be tested in comparison to Cu(NO_3_)_2_. In this context, not only other Cu types are of interest for further research, but also how porosity and crystal structure affect Cu release and antibacterial activity. In conclusion, it should be an advantage that the 5.0 wt% Cu eluate has the highest antibacterial activity and not the 10.0–15.0 wt% Cu eluate due to the possible toxicity of Cu to cells. Therefore, to determine the biocompatibility of the Cu-doped CaP supraparticles and the possible cytotoxicity of Cu, cell viability tests with hMSC-TERT cells were also performed. hMSC-TERT showed high viability over 72 h when supraparticles at a concentration of 0.01 mg mL^−1^ were applied. The use of lower Cu concentrations (≤1.0 wt%) also resulted in high cell viability in the case of increased particle concentrations (≥0.1 mg mL^−1^). From these results, the supraparticles can be classified as biocompatible with high antibacterial potentials depending on the Cu concentration, making them attractive candidates *e.g.*, as coatings for bone replacement materials. However, further *in vitro* studies are required and the extent to which osteogenic differentiation of cells is influenced by the particles needs to be investigated. For possible future bone regeneration applications, the Cu-doped supraparticle sample with 5.0 wt% Cu could be used initially, as it showed the highest antibacterial activity and at the same time lower cell toxicity compared to higher Cu concentrations (10.0–15.0 wt%). In this context, in a first experiment, 0.5 wt% of supraparticles with 5.0 wt% Cu were successfully incorporated into β-TCP coatings applied on Ti substrates (Fig. S7, ESI†). The applied coating process was high velocity suspension flame spraying (HVSFS) as described by Killinger *et al.*^63^ In this process, fine powders dispersed in a liquid can be used as feedstock, allowing the production of thin coatings (<50 μm). In addition, high cohesive/adhesive strength between the layers can be achieved due to the extremely high velocities that the particles experience during the coating process.^64^ In order to make statements about the antibacterial effect and cytocompatibility in the coating, further investigations are necessary and should be part of future research. In addition to incorporating antibacterial Cu^2+^, the supraparticles could also be tested for drug delivery to encapsulate antibiotics.

## Data availability

The data underlying this study are available in the submitted manuscript and the ESI.†

## Author contributions

Anika Höppel: investigation, visualization and writing – original draft. Olivia Bahr: investigation and writing – original draft. Regina Ebert: writing – review & editing. Annette Wittmer: investigation and writing – review & editing. Michael Seidenstücker: writing – review & editing and funding acquisition. M. Carolina Lanzino: investigation and writing – review & editing. Uwe Gbureck: writing – review & editing and supervision. Sofia Dembski: writing – review & editing, supervision and funding acquisition.

## Conflicts of interest

There are no conflicts to declare.

## Supplementary Material

RA-014-D4RA04769A-s001
