# Supplementary material for: Cu-doped calcium phosphate supraparticles for bone tissue regeneration
Source: RSC Adv. 2024 Oct 17;14(45):32839–51. doi: 10.1039/d4ra04769a (PMC11483895; doi:10.1039/d4ra04769a)
Supplement: RA-014-D4RA04769A-s001 [file RA-014-D4RA04769A-s001.pdf]

Electronic Supplementary Information

Cu-doped calcium phosphate supraparticles for bone tissue regeneration

Anika Höppel,*[a] Olivia Bahr,[b] Regina Ebert,[b] Annette Wittmer,[c] Michael Seidenstuecker, [d] M. Carolina Lanzino,[e] Uwe Gbureck [f] and Sofia Dembski[a,g]

[a] *Department Tissue Engineering and Regenerative Medicine (TERM), University Hospital Würzburg, 97070 Würzburg, Germany.*
[b] *Department of Musculoskeletal Tissue Regeneration, University of Würzburg, 97074 Würzburg, Germany.*
[c] *Medical Center University of Freiburg, Faculty of Medicine, Institute for Microbiology and Hygiene, 79104 Freiburg, Germany.*
[d] *G.E.R.N. Center of Tissue Replacement, Regeneration & Neogenesis, Department of Orthopedics and Trauma Surgery, Faculty of Medicine, Albert-Ludwigs-University of Freiburg, 79106 Freiburg, Germany.*
[e] *Institute for Manufacturing Technologies of Ceramic Components and Composites (IFKB), University of Stuttgart, 70569 Stuttgart, Germany.*
[f] *Department for Functional Materials in Medicine and Dentistry, University of Würzburg, 97070 Würzburg, Germany.*
[g] *Fraunhofer Institute for Silicate Research ISC, 97082 Würzburg, Germany.*

* *Correspondence: sofia.dembski@isc.fraunhofer.de*

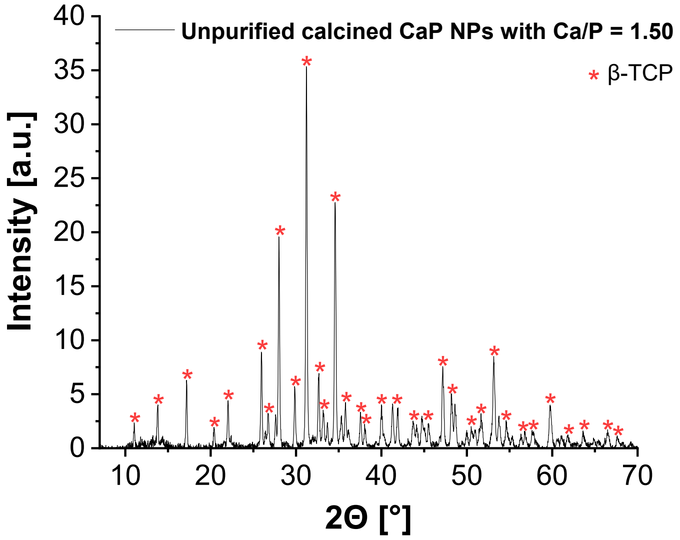

Figure S1. XRD pattern of unpurified CaP NPs with a molar ratio of Ca/P = 1.50 after calcination at 1000 °C.

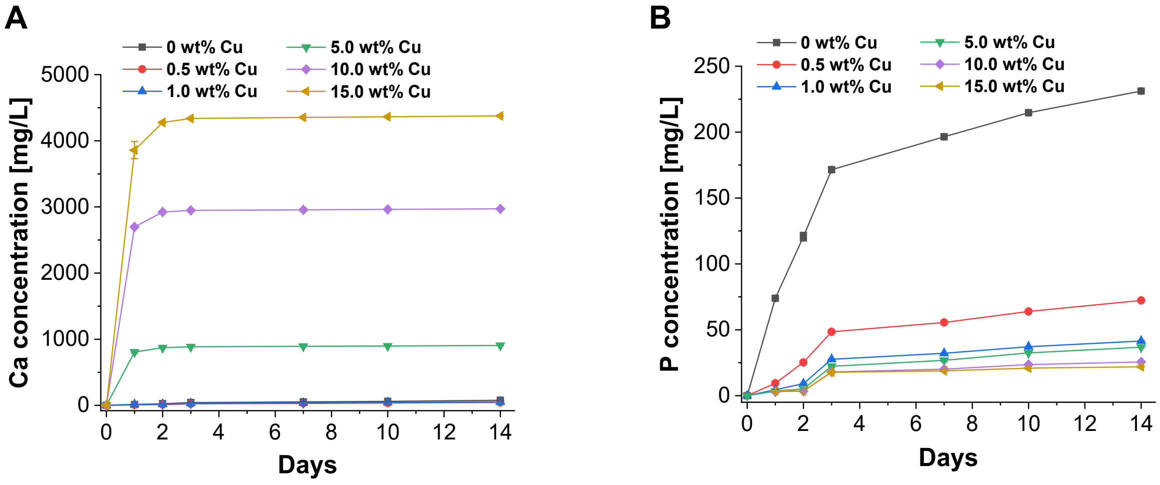

Figure S2. Ca (**A**) and P (**B**) cumulative release of Cu-doped CaP supraparticles (0 – 15.0 wt% Cu) in bi-distilled water over a period of 14 days.

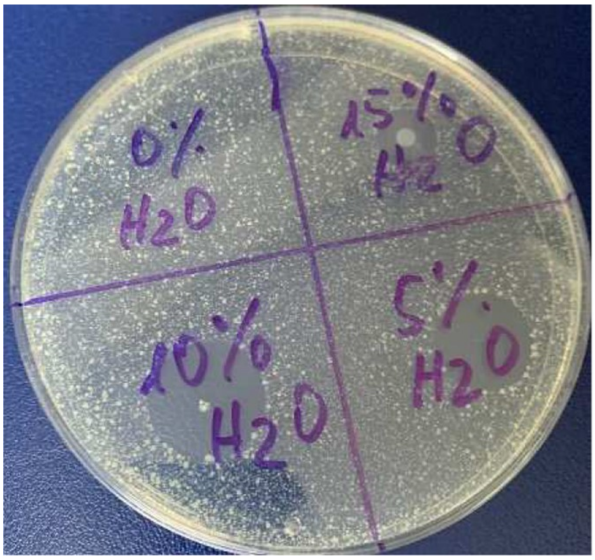

Figure S3. Results of ZOI after performing the inhibition test with *B. subtilis* using the 5.0 – 15.0 wt% Cu eluate as well as the 0 wt% Cu eluate as control.

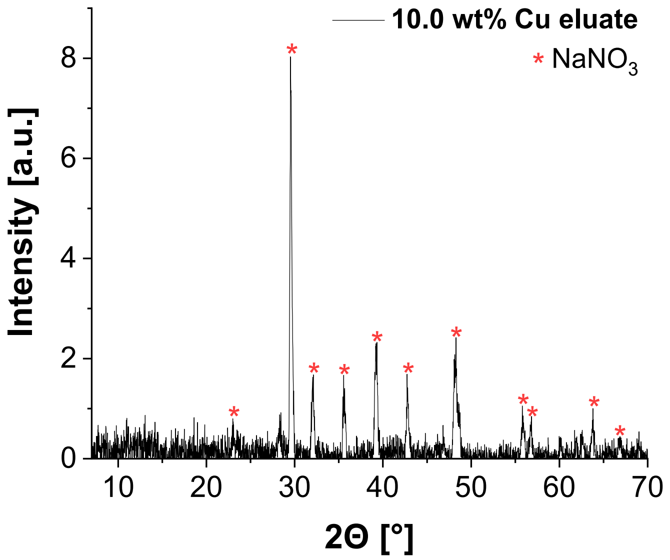

Figure S4. XRD pattern of freeze-dried 10.0 wt% Cu eluate.

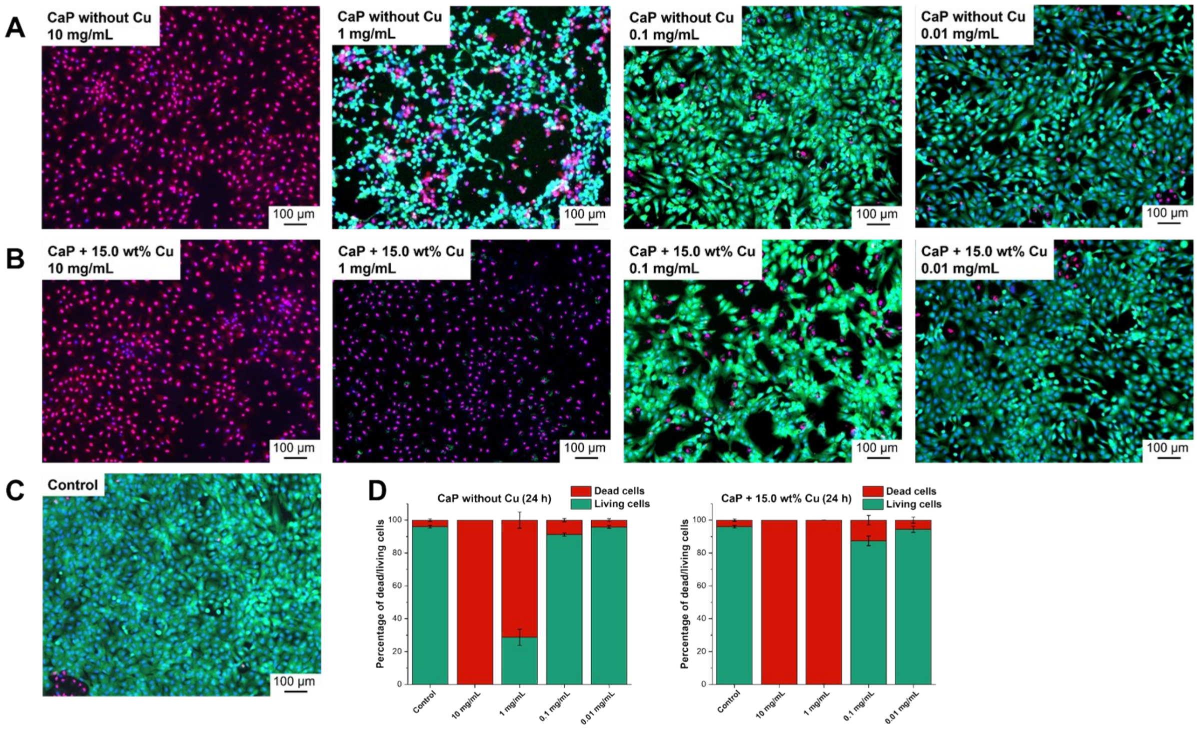

Figure S5. Live/dead staining 24 h after incubation with CaP supraparticles without (**A**) and with 15.0 wt% Cu (**B**) as well as of the control (**C**). **Green**: Fluorescence signal of living cells. **Magenta**: Fluorescence signal of dead cells. **Blue**: Fluorescence of cell nucleus. Representative images of three independent experiments are shown. (**D**) Percentage of dead/living cells of live/dead staining 24 h after incubation with undoped and Cu-doped (15.0 wt%) CaP supraparticles. The diagrams display the results of quantitative analysis of fluorescence micrographs from 3 independent experiments and 9 photos of each sample ± SEM. The analysis was performed using ZEISS ZEN 2.6 Pro software and the ZEN Intellesis module.

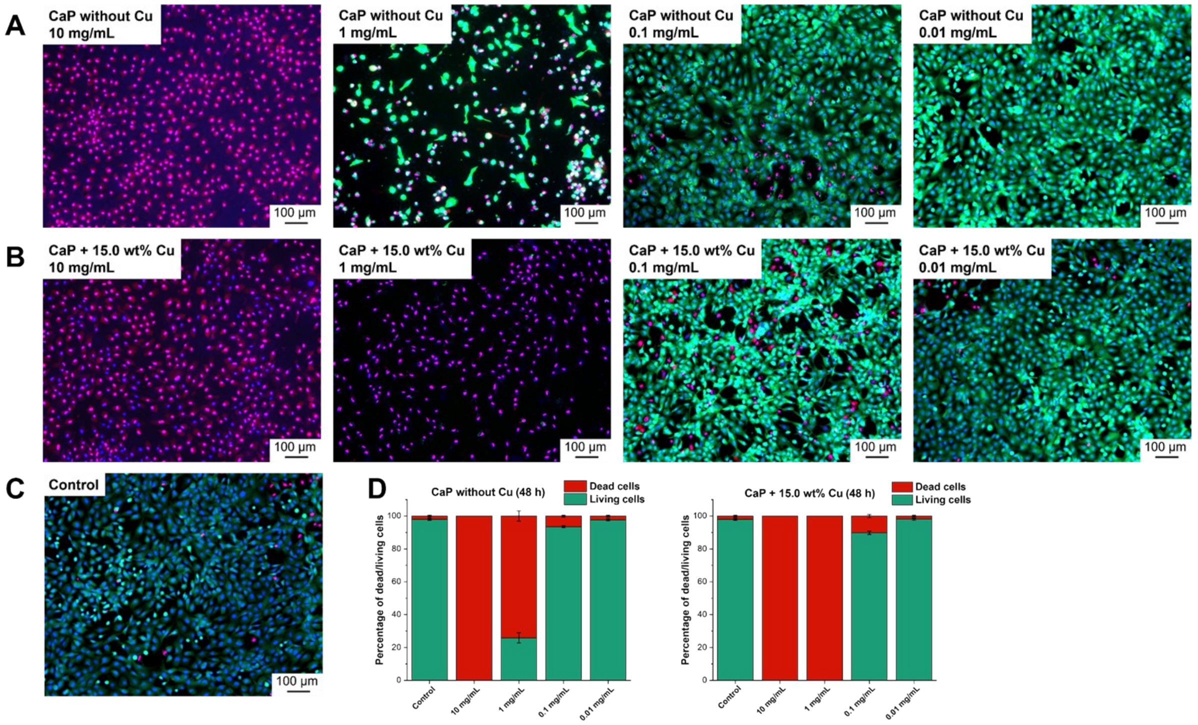

Figure S6. (A) Live/dead staining 48 h after incubation with CaP supraparticles without (**A**) and with 15.0 wt% Cu (**B**) as well as of the control (**C**). **Green**: Fluorescence signal of living cells. **Magenta**: Fluorescence signal of dead cells. **Blue**: Fluorescence of cell nucleus. Representative images of three independent experiments are shown. (**D**) Percentage of dead/living cells of live/dead staining 48 h after incubation with undoped and Cu-doped (15.0 wt%) CaP supraparticles. The diagrams display the results of quantitative analysis of fluorescence micrographs from 3 independent experiments and 9 photos of each sample ± SEM. The analysis was performed using ZEISS ZEN 2.6 Pro software and the ZEN Intellesis module.

S7. Coating and characterization: Prior to the coating process, a water-based suspension containing commercial β-TCP raw powder (5.0 wt%, Budenheim) and stabilizing agents (phosphonate-based (3.0 wt% of solid content) and hydrocolloid (2.0 wt% of total suspension)) was prepared. Subsequently, the Cu-doped CaP supraparticles (0.5 wt%) dispersed in water were introduced into the aforementioned suspension. Coating on Ti substrates (ARA-T Advance GmbH) was then performed using the HVSFS process as described by Killinger et al.[1]

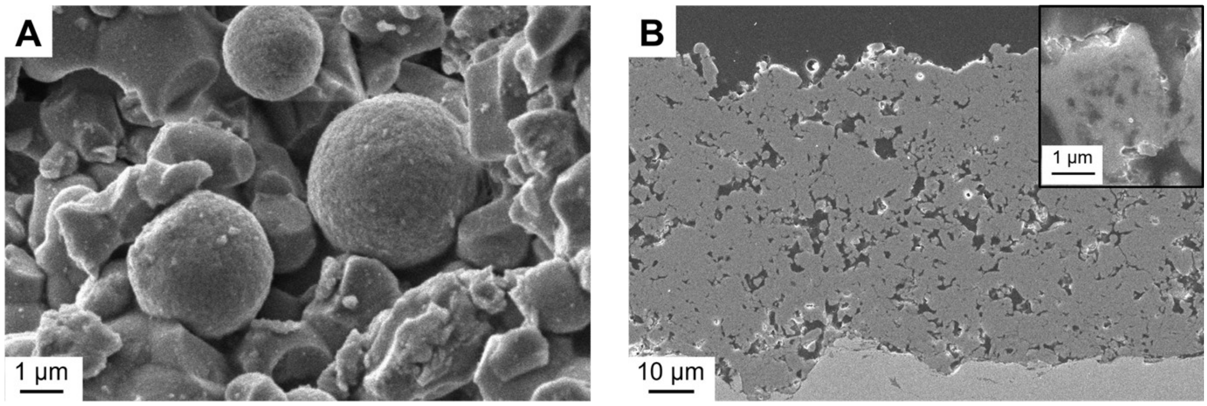

Figure S7. (**A**) SEM image of dried water suspension containing Cu-doped CaP supraparticles (0.5 wt%) and commercial β-TCP raw powder (5.0 wt%). (**B**) SEM-image of β-TCP coating with incorporated Cu-doped CaP supraparticles on Ti substrate with detail of one incorporated supraparticle (top, right).

References

1. A. Killinger, M. Kuhn and R. Gadow, *Surface and Coatings Technology*, 2006, **201**, 1922.
